# Incidence and Predictors of Canine Parvovirus Diagnoses in Puppies Relocated for Adoption

**DOI:** 10.3390/ani11041064

**Published:** 2021-04-09

**Authors:** Brian A. DiGangi, Cathlin Craver, Emily D. Dolan

**Affiliations:** American Society for the Prevention of Cruelty to Animals, New York, NY 10018, USA; cathlin.craver@aspca.org (C.C.); emily.dolan@aspca.org (E.D.D.)

**Keywords:** canine parvovirus, animal relocation, transport, vaccination, shelter medicine

## Abstract

**Simple Summary:**

Long-distance companion animal relocation programs move animals from shelters in communities with a large homeless pet population to those where there is a high demand for adoption. Basic principles of infection control and preventive care minimize the risk of unintended disease spread during program operation. This study evaluated the occurrence of canine parvovirus (CPV) diagnoses in puppies after participation in a large-scale ground transport program and the impact of shelter operational practices on such diagnoses. The rate of CPV reported in transported puppies was low, and was not different between puppies that received one or more than one vaccination prior to transport.

**Abstract:**

Animal relocation programs seek to balance the animal population and resources between source and destination communities to promote positive outcomes, though little objective evidence has been reported on their physical and behavioral implications. The objective of the current report is to describe the incidence and predictors of canine parvovirus (CPV) diagnoses in 8- to 19-week-old puppies reported by destination shelters participating in a large scale, long-distance, structured animal relocation program. The incidence of post-transport CPV diagnoses in the study population of 4088 puppies was 2.3%. The number of pre-transport vaccinations, length of stay at the source shelter, and time between pre-transport vaccination and transport was not associated with the expected difference in count of post-transport CPV diagnoses (*p* > 0.05), and was lower in those 13–17 weeks of age (IRR = 0.08, 95% CI = 0.02–0.34, *p* = 0.001), 18–19 weeks of age (IRR = 0.11, 95% CI = 0.02–0.80, *p* = 0.029), transferred in to the source shelter (IRR = 0.31, 95% CI = 0.10–0.96, *p* = 0.043), and transported in the summer season (IRR = 0.07, 95% CI = 0.01–0.53, *p* = 0.010). When basic biosecurity practices and vaccination protocols were in place, post-transport CPV cases in puppies were few, suggesting that the timing of transport should take into account factors other than the number or timing of pre-transport vaccinations.

## 1. Introduction

The relocation of healthy companion animals for adoption within the United States has become commonplace over the past decade. Although practical and experience-based guidelines and best practices for successful animal relocation have drawn increasing recognition [1,2,3], little objective evidence exists to guide operational practices and protocols. Two recent reports highlighted low rates of gastrointestinal, dermatologic, and respiratory disease in healthy dogs and cats relocated for adoption [4,5]; disease rates reported up to 30 days post-relocation were comparable to or lower than those in similar shelter populations [6,7,8,9].

There have been no published reports specifically focused on the post-transport outcomes of puppies transported by ground to date. Due to their inherent desirability, relative over-supply in some regions of the country, and under-supply but over-demand in other regions, puppies make up a large proportion of dogs relocated for adoption [10,11]. They are also at increased risk for disease as compared to adults, and are subject to the interference of maternal antibodies with vaccination response [12]. For these reasons, careful consideration should be given to understanding the factors that may influence disease susceptibility in this population.

One survey of long-distance dog transfer programs suggests a wide variability in vaccination practices required by receiving organizations (“destinations”) of sending organizations (“sources”) [10]. Pre-transport vaccination undoubtedly plays a critical role in minimizing disease incidence in populations of relocated animals, particularly for pathogens for which sterilizing immunity is expected, such as canine parvovirus (CPV). The vaccination of all dogs entering a shelter system starting at 4 weeks of age is a fundamental principle of infection control [13]; however, the timing of vaccination in relation to transportation should be carefully considered. While vaccination against core pathogens may take effect within 3–5 days of primary immunization [14,15], postponing transport for this period of time may require animals to remain at the source shelter longer than anticipated. On the other extreme, vaccination on the day of transport may not provide sufficient time for immunological response, and may result in delayed recognition or treatment in the event of an anaphylactic reaction. In the absence of objective evidence on this matter, the precise timing for vaccine administration in relation to relocation should be determined based on a risk assessment. Factors considered may include the age of the animal; their vaccination history; the protocols and conditions during transportation; the operational practices, facility design, and access to veterinary care at both the source and destination shelters; and the impact of the timing of vaccine administration on animals in place in the source and destination shelters and their communities.

The ASPCA Animal Relocation Program launched in 2014, has relocated over 150,000 animals to date, and has grown into the largest such program in the United States [16]. The current report seeks to describe the incidence of post-transport CPV in puppies participating in the ASPCA’s large-scale, long-distance, ground relocation program and the demographic characteristics of that population. An additional aim was to describe the impact of frequency of vaccination and operational practices at the source shelter on post-transport CPV diagnoses.

## 2. Materials and Methods

### 2.1. Relocation Program Standard Operating Procedures

The ASPCA Animal Relocation Program serves as a third-party transporter for partnering shelter organizations that agree to adhere to specific operational practices. Some of these practices include the vaccination on intake of all animals using modified-live virus products according to published protocols for shelter-housed animals [11], effective sanitation protocols that incorporate the use of parvocidal disinfectants, and the ability to isolate sick animals from the general population. Regardless of age, dogs accepted for relocation must have received a minimum of one modified-live virus or recombinant CPV vaccination a minimum of 24 h prior to the date of departure.

Receiving organizations are asked to complete a post-transport report (PTR) within 10 days of the transport arrival date. The report describes the current status of each animal received and notates animals for which unanticipated medical or behavioral health concerns were identified. If the PTR is not received, program staff send up to 2 e-mail reminders and, finally, contact the receiving organization by phone. Receiving shelters are also asked to contact program staff in the event of serious disease concerns, including cases of CPV that are diagnosed before or after submission of the PTR that might be attributed to an individual transport. PTR data (including individual disease issues reported outside of the PTR) are collected, and disease reports are categorized by system. Aggregate data is reviewed and analyzed on a quarterly basis.

### 2.2. Data Collection

Transport manifests for each ground transport in 2019 that included puppies between 8 and 19 weeks of age and that had an accompanying PTR were eligible for inclusion in the dataset of this report. The medical record for each transported puppy was reviewed for signalment on the transport date and vaccination history. Date and type of intake at source shelter were evaluated and categorized: “stray” includes animals relinquished as strays, abandoned, or picked up by an animal control officer; “surrender” includes animals relinquished by a known caregiver; “transfer” includes animals originally entered into the shelter system by an organization other than the relocation source partner (i.e., a source “aggregator” or “hub”); and “other” includes animals born in shelter care, returned adoptions, and those for which no intake type was recorded. PTRs were reviewed for reports of post-transport CPV cases from the destination shelter, as diagnosed by their standard operating protocols (including fecal antigen testing and/or clinical signs of infection in combination with leukopenia or direct exposure to an antigen-positive dog).

### 2.3. Statistical Analysis

All analyses were performed using Stata/IC 15.1 (StataCorpLP, College Station, TX, USA). The key outcomes and reported predictors for puppies with and without CPV diagnoses were described using frequencies and percentages. Several categorical covariates were created and described using frequencies: age (8–9 weeks, 10–12 weeks, 13–17 weeks, 18–19 weeks), intake type (owner surrender, stray + other, transfer), season of transport (winter, spring, summer, fall), the number of vaccines received (1, 2, or more), and time from last vaccination to transport (0–7 days, 8–14 days, 15 or more days). Time from intake to transport was described as the total number of days plus one to include animals transported on the day of intake.

A multilevel, mixed-effects Poisson model with robust standard errors was fit as the main model with clustering by membership in a litter (with all singleton puppies treated as one litter) to account for variance. The variables described above were included as main effects in the model, and were retained for their clinical relevance and biological plausibility to the research question. The outcome was whether or not a CPV diagnosis was reported post-transport. An interaction was included between the number of vaccines received (categorical) and the time from intake to transport (continuous). *p*-Values < 0.05 were considered significant.

## 3. Results

### 3.1. Study Population

A total of 4088 puppies were eligible for inclusion, with a median age of 11 weeks (IQR = 9–13 weeks). The study population included 2017 intact females (49.3%), 1825 intact males (44.6%), 118 spayed females (2.9%), and 128 neutered males (3.1%). Counting singleton puppies as one litter (*n* = 644), the study population was comprised of 835 litters (median litter size = 3; IQR = 2–5, excluding singleton litter). Puppies arrived at the source organization by transfer from regional partners (*n* = 1729; 42.3%), surrender by a caretaker (*n* = 1356; 33.2%), stray (*n* = 963; 23.6%), or other form of intake (*n* = 40; 0.98%) (Table 1). Puppies described in this report were transported between 3 January and 29 December, 2019 through 426 individual source–destination transport partnerships; 119 of these transports included animals from multiple sources on a single vehicle. Puppies originated from 24 different source organizations in states in the Southeastern (*n* = 23) and Western (*n* = 1) regions of the United States. Puppies were relocated to a total of 28 different destination organizations in states in the Midwestern (*n* = 15), Northeastern (*n* = 10), Western (*n* = 2), and Southeastern (*n* = 1) regions of the United States.

A total of 94 individual CPV diagnoses occurring across 33 different transports were reported by destination organizations during the study period, for an incidence of 2.3%. In 16 cases, CPV diagnoses were made in two or more co-housed puppies from the same litter. During the study period, five adult dogs were also diagnosed with CPV occurring across four different transports. In two of those transports (including three adult CPV diagnoses), transports included both adult dogs and puppies, but such diagnoses were only reported in adults.

### 3.2. Animal and Operational Factors

**Figure 1:**
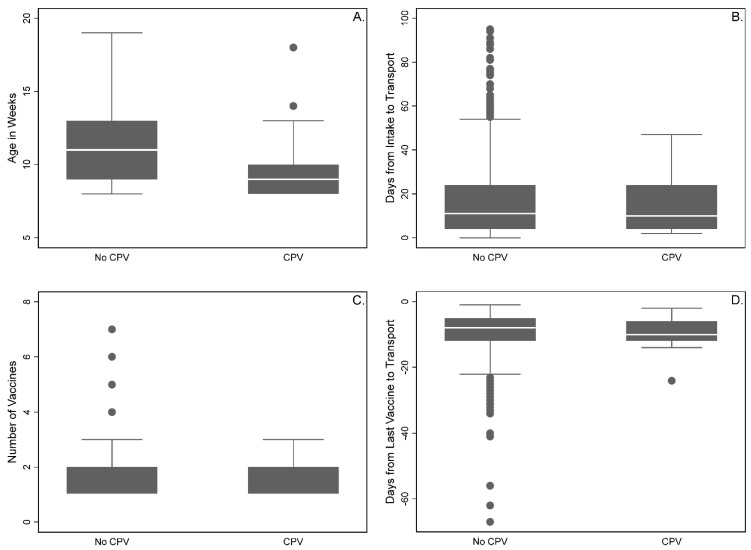
Box and whisker plots of the (**A**) age in weeks, (**B**) days from intake to transport, (**C**) number of vaccines, and (**D**) days from last vaccine to transport for 94 puppies with (CPV) and 3994 puppies without (No CPV) post-transport canine parvovirus diagnoses.

Controlling for all variables in the model and accounting for clustering by litter, compared to puppies less than 10 weeks of age at the time of transport, the expected difference in the count (i.e., incidence rate ratio (IRR)) of post-transport CPV diagnoses was lower in those 13–17 weeks of age (IRR = 0.08, 95% CI = 0.02–0.34, *p* = 0.001) and 18–19 weeks of age (IRR = 0.11, 95% CI = 0.02–0.80, *p* = 0.029) (Table 2; Figure 1). There was no such difference in puppies less than 10 weeks of age compared to those 10–12 weeks of age (*p* = 0.530). There were also no expected differences in the count of post-transport CPV diagnoses based on sex (*p* = 0.495) or neuter status (*p* = 0.067).

Controlling for all variables in the model and accounting for clustering by litter, compared to puppies that entered the source shelter as stray + other, the expected difference in the count of post-transport CPV diagnoses was lower in those that were transferred in to the source shelter (IRR = 0.31, 95% CI = 0.10–0.96, *p* = 0.043). In addition, as compared to puppies that were transported in the fall season, the expected difference in the count of post-transport CPV diagnoses was lower in those that were transported in the summer season (IRR = 0.07, 95% CI = 0.01–0.53, *p* = 0.010).

All puppies were transported a median of 10 days after intake to the source partner (IQR = 4–24; Figure 1). There was no expected difference in the count of post-transport CPV diagnoses based upon number of days from intake to transport (*p* = 0.121).

### 3.3. Vaccination History

**Figure 2:**
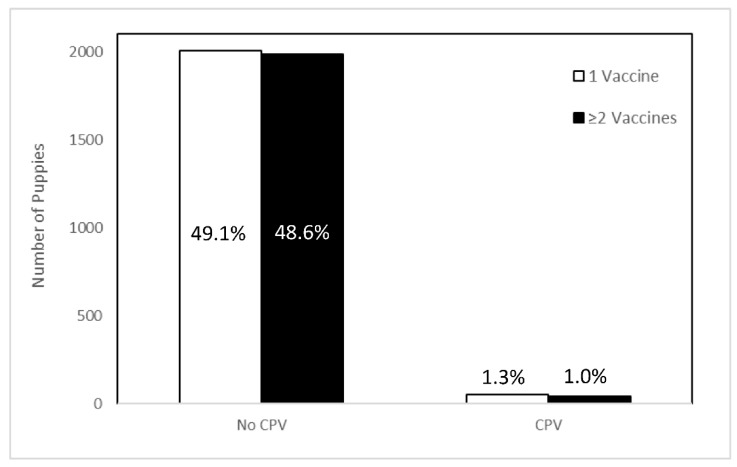
Number and percentage of puppies between 8 and 19 weeks of age receiving one (white bars) or ≥2 (black bars) modified-live virus vaccinations against canine parvovirus (CPV) prior to transport for whom diagnoses of CPV were reported (*n* = 94) and were not reported (*n* = 3994) after relocation. There was no difference in the expected count of CPV diagnoses between vaccination groups.

Relocated puppies received a median of one modified-live virus vaccination against CPV prior to transport (IQR = 1–2; Figure 1) and the final vaccination prior to transport was given at median −8 days (IQR = −12 to −5; Figure 1). Puppies with reported diagnoses of CPV received a median of one modified-live virus vaccination against CPV prior to transport (IQR = 1–2) and the final vaccination prior to transport was given at median day −10 (IQR = −12 to −6). Puppies without reported diagnoses of CPV received a median of one modified-live virus vaccination against CPV prior to transport (IQR = 1–2) and the final vaccination prior to transport was given at median day −8 (IQR = −12 to −5). There was no significant interaction between the number of vaccines received and the number of days from intake to transport on the expected count of CPV cases (*p* = 0.175; Figure 2).

Controlling for all variables in the model and accounting for clustering by litter, there was no expected difference in the count of post-transport CPV diagnoses in puppies that received one vaccination prior to transport as compared to those that received two or more vaccinations (IRR = 0.25, 95% CI = 0.04–1.46, *p* = 0.125) (Table 2). Similarly, there was no expected difference in the count of post-transport CPV diagnoses in puppies that were last vaccinated 8–14 days (IRR = 1.5, 95% CI = 0.57–4.12, *p* = 0.396) or 15+ days (IRR = 0.68, 95% CI = 0.12–3.92, *p* = 0.668) prior to transport as compared to those that were vaccinated 0–7 days prior to transport.

## 4. Discussion

This report identified a low rate of post-transport CPV diagnoses in puppies; whether or not a CPV diagnosis was made was not influenced by the number of pre-transport vaccinations, time from intake, or time from last vaccination to transport. Compliance with current best practices in animal sheltering and animal transport appear to be an effective means of minimizing infectious disease risk in relocated puppies. To the authors’ knowledge, this is the first report to characterize the incidence of post-transport CPV diagnoses in puppies relocated long-distance for adoption. A previous report described reports of gastrointestinal disease in 5 of 280 (2%) adult dogs and puppies as reported by adopters post-relocation; one of those dogs was known to have a diagnosis of CPV [4]. Another report identifying enteropathogens in 100 dogs shortly after admission to a Florida animal shelter identified CPV in 2% of adult and juvenile (<6 months) dogs both with and without diarrhea, though the possibility of detection of vaccine virus in some of these cases could not be ruled out [17]. Factors such as lack of protective immunity, intestinal parasitism, overcrowding, and stress are thought to predispose puppies to parvoviral infection [18], and are commonly presumed to be present in shelter populations. The overall low occurrence of CPV diagnoses in these three reports suggests that such conditions are not universally present in animal shelter populations, and/or other factors may be just as—if not more—important in assessing risk for CPV infection in puppies.

In the study sample, there was no difference in the expected count of post-transport CPV diagnoses between puppies vaccinated once or two or more times prior to transport, controlling for age. Similarly, the timing of vaccination prior to transport was not statistically significant in the reported model, downplaying the importance of precise timing of administration to allow for measurable immunologic effect. As mentioned previously, although it may be ideal to extend the interval between vaccination and transport in order to allow for a primary immune response, remaining at the source shelter is not without risk. Furthermore, when the risk of exposure during the transport itself is extremely low, it may be counterproductive. Taken together, these data lend support to the notion that factors other than the number of vaccinations received prior to transport are perhaps more important than—or at least, equivalent to—factors such as receipt of a modified-live virus vaccination and strong biosecurity protocols to minimizing the risk of disease exposure and transmission in populations of relocated puppies. Importantly, these data also demonstrate that there is no additional benefit in reducing post-transport CPV cases by maintaining puppies at a source shelter solely for the purpose of additional vaccinations.

Active management of length of stay of shelter animals is a key component of maintaining both physical and behavioral health; stays longer than 14 days are generally considered long-term [3]. In dogs, increasing length of stay has been associated with increased exposure to canine influenza virus and increased frustration behaviors [19,20]. Although it is rational and prudent to assume that a longer duration of exposure to a shelter environment with a concentrated animal population and high turnover would result in increased exposure to and transmission of CPV, the current report found no expected difference in the count of post-transport CPV diagnoses in puppies based on the number of days between intake and transport. This may be explained by the relatively short-term stay of relocated puppies (median of 11 days) and/or the operational protocols at the source shelters. Program partners are required to utilize sanitation protocols that encompass both cleaning and disinfection steps and disinfectant products with independently demonstrated efficacy against non-enveloped viruses. In addition, source partners must ensure a means of physical separation between any animals housed at the source shelter that have clinical signs of infectious disease and those that are clinically healthy (i.e., a designated isolation ward or plan to manage potentially infectious animals off-site). Similar biosecurity protocols are enforced throughout the transportation itself and these factors, in combination with the vaccination protocols described above, likely contribute to the overall low rate of disease described.

In the study sample, fewer cases of CPV were predicted in older puppies compared to those less than 10 weeks of age when controlling for all other variables in the model. This is not unexpected, and is likely attributed to the proportional decrease in maternal antibody levels with age. Notably, this effect was not predicted in puppies 10–12 weeks of age, emphasizing the variable nature of maternal antibody levels and their natural decline [21]. Fewer cases of CPV were also predicted in puppies transferred into the source shelter as compared to stray intakes. This effect could be attributed to care provided to those animals by the organization of origin prior to presentation to the relocation source shelter. Many originating organizations are foster-based, meaning animals in their care have less exposure to the high-density and high-turnover population found in traditional animal shelters and, therefore, lower risk of exposure to infectious disease. A third factor associated with a decreased rate of CPV cases was the transport season. Fewer cases were predicted for summer transports as compared to those conducted during the fall months. The reason for this relationship is unclear, and previous reports of similar populations of dogs have described both a peak in CPV incidence in the summer months [22] as well as a lack of seasonal variation [23]. Given these discrepancies, it seems likely that seasonality is of minor importance when assessing risk of CPV infection.

This study has several limitations that should be considered when reviewing the findings. First, CPV cases were reported by destination organizations, and method or accuracy of diagnoses were not standardized or verified. The diagnostic criteria used are in accordance with typical shelter operational protocols, and were intentionally broad to capture all likely cases. The possibility of undiagnosed or unreported cases cannot be ruled out, but program personnel maintain frequent and regular contact with shelter partners and, given the population health implications and resource requirements to care for puppies diagnosed with CPV, the authors consider it extremely unlikely that any cases known to the destination organization were unreported. Similarly, destination organizations report a short duration of stay between arrival at the destination and adoption (mean of 8 days) [11], so it is also possible that some adopted puppies may have received a CPV diagnosis post-adoption that was not reported back to the destination organization.

The precise timing of receipt of the PTR and diagnosis of CPV was also not tracked, and such timing likely varied widely. It is possible that some reports were returned prior to establishing a CPV diagnosis while others were returned comparatively late, allowing for factors other than those immediately associated with transport to influence the likelihood of exposure, transmission, and development of CPV. As reported above, 17% of CPV cases were diagnosed in co-housed littermates, which suggests that exposure and susceptibility factors prior to transport were at play. Clustering by source agency and geographic location was investigated in the current report, but did not improve the performance of the model (data not shown). Although it would be a useful data point to track from a program operational perspective moving forward, the current data do not allow for further objective analysis of the likely point of exposure or transmission of CPV in affected puppies.

The historical vaccination data described is also subject to some variability. Although all partnering organizations adhere to the same basic principles and protocols (i.e., modified-live virus vaccination of all puppies starting at 4 weeks of age, repeated every 2 weeks while shelter-housed, until 20 weeks of age), vaccine manufacturer, handling, and administration practices all vary by individual organization. For these reasons, it is not possible to estimate the likelihood that vaccination led to immunization in any case. The data regarding the timing of the last vaccination prior to transport should also be considered with caution. Program operating protocols call for the vaccination of puppies no longer than 14 days prior to transport, however, the range of “final” pre-transport vaccination days (day −67 to −1) suggests that these protocols were not followed in all cases or were not reported accurately. If the former, the authors hypothesize that the incidence of CPV diagnoses would have been higher than reported; if the latter, meaning that additional vaccinations were indeed given just not recorded, this may have impacted the analyses regarding the timing of vaccination in relation to transport.

Regarding the duration of shelter stay prior to transport, it is important to recognize that while the intake date as recorded by the source partner was analyzed, this may not represent the precise date of intake into the sheltering system for each puppy. Some source organizations serve as “aggregators” or “hubs” and receive animals from other organizations in order to prepare and send them on a scheduled transport. Additionally, while 42% of the puppies included were known to be transferred into the source organization for the purpose of relocation, it was not possible to confirm if this was uniformly recorded across all records. Across the dataset as a whole, it is possible that the intake date and type on the record may reflect either the animals’ initial point of entry into the shelter system or merely the date of admission to an organization for a scheduled transport. Similarly, it is likely that some puppies spent time in private foster homes for some portion of their duration of shelter stay. These data were unavailable for analysis, and may have altered the level of disease risk experienced by or attributed to each puppy in relation to duration of shelter stay.

## 5. Conclusions

Reports of CPV diagnoses after relocation via a long-distance ground transport were uncommon. The number of vaccinations, timing of vaccination relative to transport, and timing from intake to transport were not related to the frequency of post-transport CPV diagnoses in this exploratory analysis. These data suggest that the precise timing of vaccine administration in relation to relocation would better be determined based on a broader risk assessment which includes protocols and conditions during transportation; the operational practices, facility design, and access to veterinary care at both the source and destination shelters; and the impact of the timing of transport on animals in place in the source and destination shelters and their communities. Based on these findings, as long as a minimum of one modified-live virus CPV vaccination is appropriately administered, the authors believe there is no benefit to maintaining puppies selected for relocation at the source shelter for the purpose of additional vaccinations; completion of the primary vaccination series should continue after arrival at the final destination and/or adoption. Adherence to basic principles of biosecurity and preventive care were effective means of minimizing post-transport CPV diagnoses; organizations wishing to minimize the risk of infectious disease transmission and maximize the life-saving benefits of animal relocation programs should strive to meet or exceed these practices in order to achieve sustained success.

## Figures and Tables

**Table 1:** Characteristics of 4088 puppies transported in a long-distance animal relocation program in the continental United States in 2019. CPV = canine parvovirus

Characteristic	CPV Reported (*n* = 94) N (%)	No CPV Reported (*n* = 3994) N (%)
Median age (weeks)	11	11
Sex		
Male	51 (54.3)	1902 (47.6)
Female	43 (45.7)	2092 (52.4)
Neuter status		
Intact Neutered	87 (92.6) 7 (7.4)	3755 (94.0) 239 (6.0)
Intake category		
Stray	38 (40.4)	925 (23.2)
Surrender	28 (29.8)	1328 (33.2)
Transfer	28 (29.8)	1701 (42.6)
Other	0 (0)	40 (1.0)
Season of transport		
Winter (1 January–18 March, 22–31 December)	16 (17.0)	912 (22.8)
Spring (19 March–19 June)	38 (40.4)	1311 (32.8)
Summer (20 June–21 September)	14 (14.9)	1086 (27.2)
Fall (22 September–21 December)	26 (27.7)	685 (17.2)
No. of CPV vaccinations prior to transport		
1	52 (55.3)	2007 (50.3)
≥2	42 (44.7)	1987 (49.7)
Time from last vaccination to transport (days)		
0–7 8–14 15+	43 (45.7) 42 (44.7) 9 (9.6)	1949 (48.8) 1730 (43.3) 315 (7.9)
Median (IQR) time from intake to transport (days)	12 (5–25)	11 (5–25)

**Table 2:** Mixed effects Poisson regression model for 4088 puppies transported in a long-distance animal relocation program in the continental United States in 2019.

Category	Incidence Rate Ratio	*p*-Value	95% Confidence Interval
Age (weeks) on transport			
<10 10–12 13–17 18–19	Ref. 0.73 0.08 0.11	Ref. 0.530 0.001 0.029	Ref. 0.27–1.97 0.02–0.34 0.02–0.80
Sex			
Male Female	Ref. 0.92	Ref. 0.495	Ref. 0.72–1.17
Neuter status			
Intact Neutered	Ref. 2.13	Ref. 0.067	Ref. 0.95–4.78
Source			
Stray Surrender Transfer	Ref. 0.38 0.31	Ref. 0.092 0.043	Ref. 0.13–1.17 0.10–0.96
Season			
Fall Spring Summer Winter	Ref. 0.52 0.07 0.39	Ref. 0.341 0.010 0.163	Ref. 0.14–1.98 0.01–0.53 0.10–1.47
Days from intake to transport	0.88	0.121	0.75–1.03
Number of MLV DA_2_PP * vaccines prior to transport			
1 ≥2	Ref. 0.25	Ref. 0.125	Ref. 0.04–1.46
Number of MLV DA_2_PP vaccines prior to transport XDays from intake to transport	1.12	0.175	0.95–1.32
Days between last vaccine and transport			
0–7 8–14 ≥15	Ref. 1.53 0.68	Ref. 0.396 0.668	Ref. 0.57–4.12 0.12–3.92

* MLV DA_2_PP = modified live-virus distemper-adenovirus-2-parainfluenza-parvovirus
